# Optical Coherence Tomography in Glaucoma

**DOI:** 10.5005/jp-journals-10008-1099

**Published:** 2012-10-16

**Authors:** P Sathyan, Shilpa Shilpa, Anitha Anitha

**Affiliations:** 1 Glaucoma Services, Aravind Eye Hospital, Coimbatore, Tamil Nadu, India; 2 Glaucoma Services, Aravind Eye Hospital, Coimbatore, Tamil Nadu, India; 3 Glaucoma Services, Aravind Eye Hospital, Coimbatore, Tamil Nadu, India

**Keywords:** AS-OCT, RNFL OCT, Glaucoma diagnosis.

## Abstract

The optic disk and the RNFL are the principal sites of apparent glaucomatous damage which precedes glaucomatous visual field alterations. RNFL defects are known to precede detection of visual field defects by approximately 6 years. Accurate early detection and monitoring of ONH and RNFL defects has become the prime focus of effective management of glaucoma. Optical coherence tomography employs low-coherence interferometry to obtain cross-sectional images of the ocular tissues.

This review attempts to critically analyse the applications of both, anterior and posterior segment OCT in glaucoma management.

Glaucoma is an optic neuropathy with characteristic optic nerve appearance and visual field loss for which elevated intraocular pressure (IOP) is one of the main risk factors.^1^ This characteristic optic nerve appearance results from structural glaucomatous changes which usually precede functional deterioration (visual field loss). Worldwide, glaucoma is the most common cause of irreversible blindness and the second leading cause of blindness.^2^ The optic disk and the RNFL are the principal sites of apparent glaucomatous damage which precede glaucomatous visual field alterations. In 60% of reported cases, retinal nerve fiber layer (RNFL) defects preceded detection of visual field defects by approximately 6 years.^3^ Accurate early detection and monitoring of optic nerve head (ONH) and RNFL defects have become the prime focus of effective management of glaucoma.

## INTRODUCTION

Optical coherence tomography (OCT) is an imaging technology that employs low-coherence interferometry to obtain cross-sectional images of the ocular tissues. The principle of OCT is analogous to that of ultrasonography, but uses light instead of sound to acquire high resolution images of the ocular structures. A beam of light is shone on the eye and reflections returning from the structures are analyzed to produce real time images.

## PRINCIPLES OF OCT

A light source is directed to a partially reflecting mirror that splits the light into two beams: One is directed toward a mirror placed at a known distance (reference mirror) and the other is directed toward the eye, from where it will reflect back. The back-reflected light from the eye is combined with the back-reflected light from the reference mirror and coherent light is compared. Interference is produced when two light pulses coincide. The reference mirror is then moved so that the time delay of the reference light pulse can change accordingly and therefore other intraocular structures can be measured. The laser beam is passed throughout the tissue and a series of scans are obtained to produce a two-dimensional map.

## APPLICATION OF OCT IN GLAUCOMA

### Anterior Segment-OCT

Anterior segment OCT (AS-OCT) uses light of longer wavelength (1310 nm) to obtain images of the anterior segment. Light-induced damage of the retina at this high wavelength is prevented by the absorption of light by the water in the ocular tissues.

Applications of AS-OCT

**Fig. 1:**
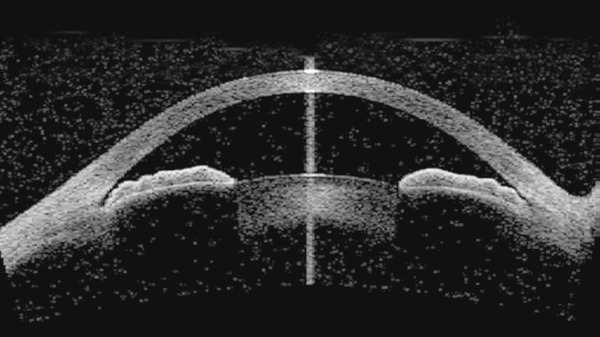
AS-OCT showing 180° narrow angles

1. Evaluation of angle structures. It causes minimal distortion of the angle anatomy because no visible light is shone on the eye and there is no pressure on the globe.
2. AS-OCT provides detailed calculations of parameters, such as angle opening distance, angle recess area, and the trabecular―iris space area, thus introducing new levels of precision for approaching PAC disease (Fig. 1).
3. To evaluate the effect of laser peripheral iridotomy and other interventions on the angle anatomy, which helps the clinician plan further interventions if necessary.
4. Assessment of bleb morphology and patency of ostium post-filtering surgery helps the clinician to plan further interventions as necessary.

### Posterior Segment-OCT

Posterior segment OCT uses light of 830 nm to obtain images of the posterior segment structures, such as ONH, retinal nerve fiber layer and macula. It has software that facilitates image acquisition, storage, retrieval and analysis. Several topographic ONH parameters are automatically calculated and are reported along with a color-coded map and ONH topographic map.

**Fig. 2:**
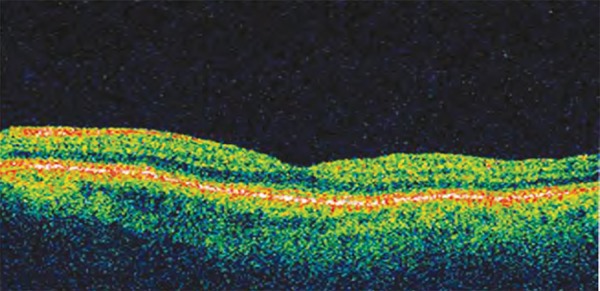
Stratus OCT

**Fig. 3:**
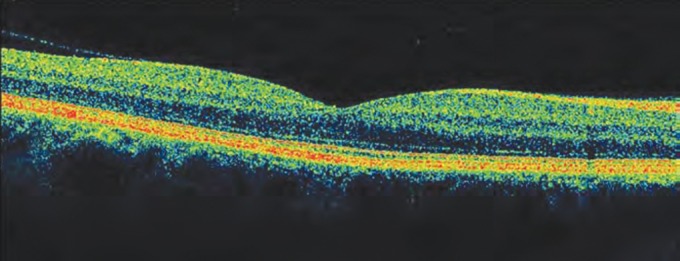
Spectral domain OCT

OCT has evolved through different modifications from OCT1 to fourier domain/spectral domain OCT. The newer method of spectral domain (SD) OCT has higher speed and resolution than its predecessor, Stratus OCT (Figs 2 and 3). Spectral-domain OCT also captures three-dimensional images of optic disk and surrounding tissue components. Three of the commonly used SD-OCT devices are the Spectralis (Heidelberg Engineering, Dossenheim, Germany), the Cirrus (Carl Zeiss Meditec, Dublin, CA) and the RTVue (Optovue Inc, Fremont, CA).

Axial resolution of spectral-domain (SD) OCT is twice higher (5-7 microns) than stratus OCT (approximately 10 microns). The SD OCT instruments can acquire B-scans 45 to 130 times faster than Stratus OCT and multiple B-scans can be acquired at the same location, when averaged results in a speckle-noise-reduced image with clearly distinguishable boundaries between retinal layers. It also has three-dimensional (3-D) eye tracking system (Spectralis HRAOCT; Heidelberg Engineering, Heidelberg, Germany) that reduces motion induced artifacts.

### Interpretation of OCT (FIGS 4 to 8)

Quality Assessment

1. Appropriate centration of the peripapillary circular scan is essential for accurate measurements of RNFL thickness.
2. Signal strength value of the scan should be greater than 5.
3. Homogeneity of the RNFL scan is important since loss of reflectivity can affect the overall quality.

The figure shows normative data of Stop-Light color scheme scan with areas of defect in any given patient (normative population). This helps the physician to easily read the chart without much difficulty.

Macular Nerve Fiber Layer Assessment

OCT scans of the macula involve a strategy of six intersecting lines that intersect at the foveal center. Assessment of the macular region in glaucoma is necessary because over 50% of retinal ganglion cells lie in the macular region and hence it is the ideal region to detect early cell loss.

In macular thickness analysis by Stratus OCT central 3 mm of the posterior pole have the highest scan density compared with the outer (3 to 6 mm) concentric ring.

The ganglion cell complex (GCC) which includes: (1) the retinal nerve fiber layer (RNFL), (2) the ganglion cell layer (GCL) and (3) the inner-plexiform layer (IPL) becomes thinner due to ganglion cell loss in glaucoma. Spectral OCT (RTVue) measures the thickness of GCC in macular region and gives analysis compared to an extensive normative database.

GCC scan data is displayed as thickness map of GCC layer. This map is color-coded where thicker regions are displayed in hot colors (yellow and orange) and thinner regions in cooler colors (blue and green). GCC map for a normal eye shows a bright circular band surrounding the macula representing thick GCC as depicted below.

### Clinical Applications of OCT

1. *Retinal nerve fiber layer analysis:* RNFL thickness measurement is graphed in a TSNIT orientation and compared to age matched normative data. Decreased RNFL thickness represents glaucoma.
2. *ONH analysis:* Disk margins are objectively identified by using signal from and of RPE. Key parameters include cup to disk ratio and horizontal integrated rim volume.
3. *Macular thickness analysis:* Thinning of macula may reflect glaucomatous loss.
4. A recent software upgrade of Stratus OCT (Stratus OCT Version 5.0) has included the glaucoma progression analysis to evaluate the association between average RNFL thickness and age.

### Advantages of OCT

1. Easy to operate.
2. It has the best resolution among all the imaging devices.
3. Has a rapid image acquistion time.
4. Being a noncontact technique, images can be obtained without causing undue discomfort to the patient.
5. Qualitative and quantitative data can be collected and analyzed in an objective and reproducible way.
6. It is the only technology capable of imaging the optic nerve head, retinal nerve fiber layer and macula.
7. Can obtain posterior segment images without pupillary dilatation.

**Fig. 4:**
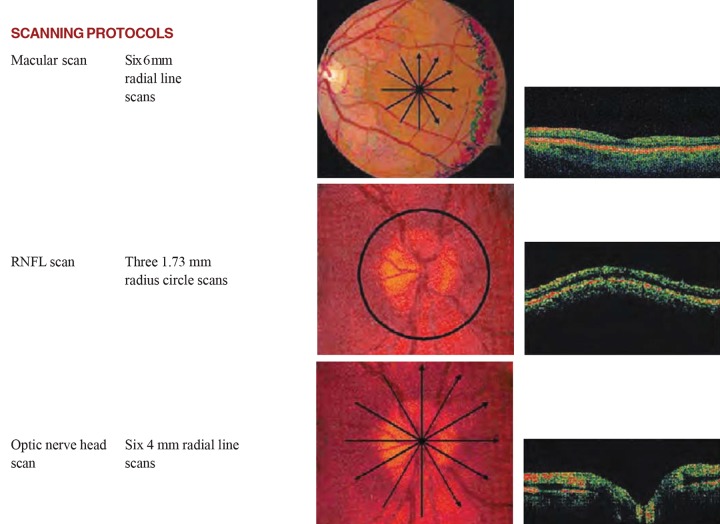
Scanning protocols

**Fig. 5:**
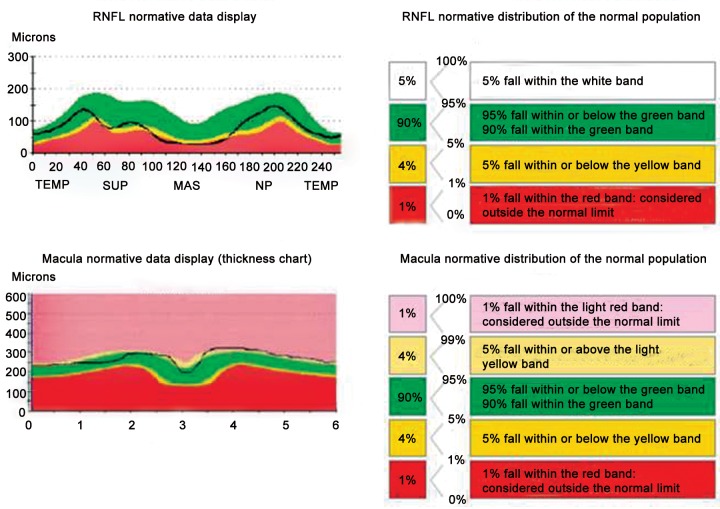
Statistical significance and normative database

**Fig. 6:**
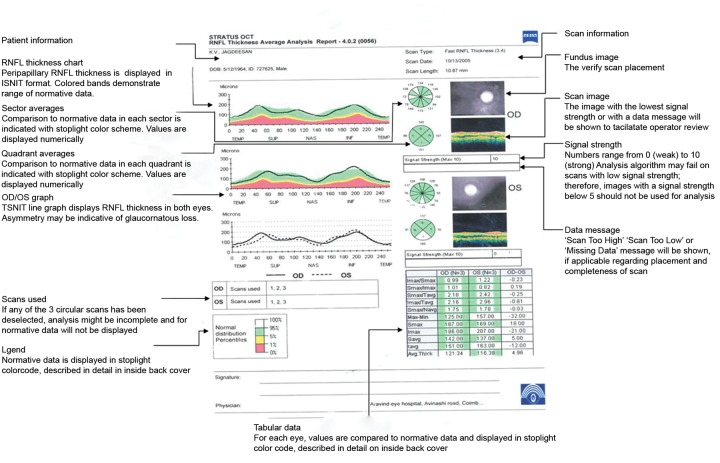
RNFL thickness chart

**Fig. 7:**
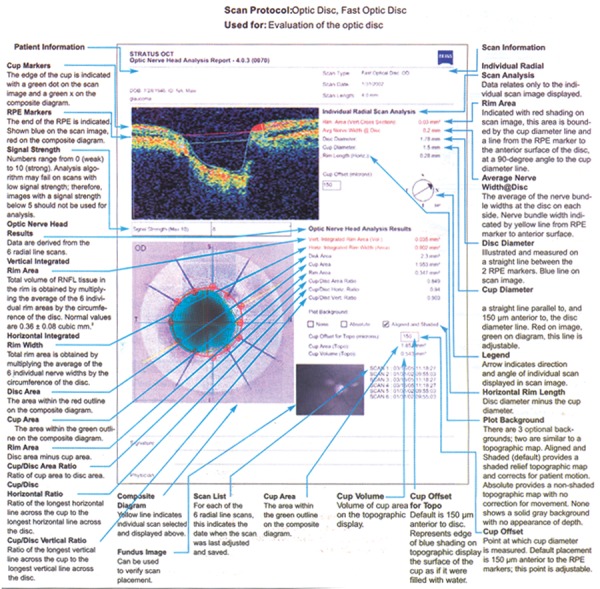
Optic nerve head analysis chart

**Fig. 8:**
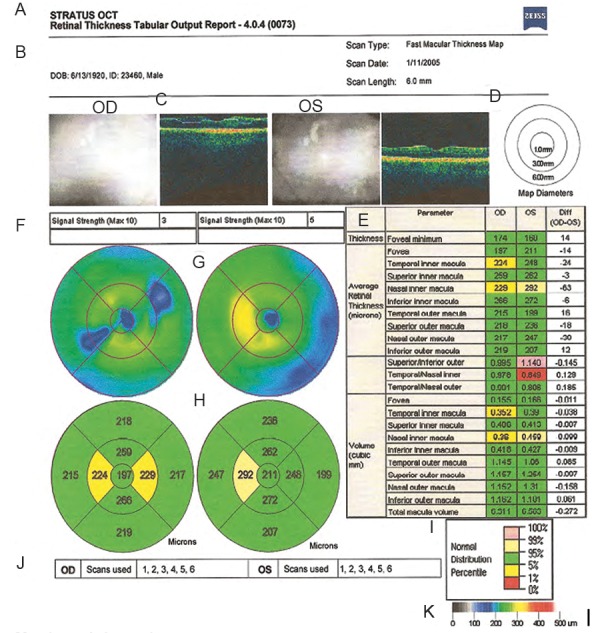
Macular thickness analysis

### Precision of OCT in Early Diagnosis of Glaucoma

Various studies on OCT^4-8^ have shown:

- Measurement of RNFL thickness with OCT has been reliable in discriminating normal from glaucomatous eyes.
- OCT has good sensitivity and specificity for differentiating normal from glaucomatous eyes. A study by Chang et al showed Stratus OCT sensitivity and specificity for average RNFL abnormal at the 5% level were 80 and 94% respectively and at 1% level were 61 and 100% respectively. Cirrus OCT sensitivity and specificity for average RNFL abnormal at the 5% level were 83 and 88% respectively and at the 1% level were 65 and 100% respectively.^9^

### Limitations of OCT

1. AS-OCT has a poor ability to show the details of ciliary body and the posterior surface of the iris ( since the posterior pigment epithelium of the iris and the ciliary epithelium block the passage of infrared light). Therefore AS-OCT can not detect cyclodialysis clefts and ciliary body tumors.
2. Image quality of superior and inferior quadrants of the angle with AS-OCT is suboptimal due to interference from the eyelids.
3. Landmarks, such as scleral spur and Schwalbe’s line are not always clearly visible as with UBM, so quantitative measurements of angle width may not be very accurate.
4. Automatic demarcation of the optic disk borders by the machine may be inaccurate in cases of parapapillary atrophy, which would confound the interpretation of optic disk topography. This may limit the ability of OCT to detect the progression of glaucomatous optic disk damage.
5. It is possible that localized NRR/optic cup changes would be missed by the interpolation algorithm.
6. Depends on the skill of the operator.
7. Poor quality of images in dense media opacities.
8. Difficult in uncooperative patients.
9. Expensive instrumentation.

## CONCLUSION

Medicine and technology are advancing hand in hand to provide quality health care. Technology innovation and improvement will continue to impact health services.

OCT is a new technology. Any new technology introduces both difficulties and opportunities. The lack of large scale normative database is perhaps the greatest issue in interpretation of OCT results at this point of time. These issues must be resolved before OCT can be accepted for widespread clinical use in glaucoma. Apart from this note of caution the potential utility of OCT as a glaucoma diagnostic tool is extremely high as, adequate data exist to evaluate the patients in conjunction with other clinical parameters. A patient can be followed over time, using his or her own baseline. The two eyes of the patient can be compared for asymmetry, and a single eye can be examined for focal or sectoral NFL thinning.

All pieces of the glaucoma puzzle must be put together in order to care appropriately for the patient. The clinician must correlate clinically with IOP, ONH and NFL appearance, visual field data, as well as quantitative data contributed by technology, to detect glaucoma and its progression.
